# Feasibility and acceptability of a multicomponent, group psychological intervention for adolescents with psychosocial distress in public schools of Pakistan: a feasibility cluster randomized controlled trial (cRCT)

**DOI:** 10.1186/s13034-022-00480-z

**Published:** 2022-06-21

**Authors:** Syed Usman Hamdani, Zill-e Huma, Asad Tamizuddin-Nizami, Um-ul Baneen, Nadia Suleman, Hashim Javed, Aiysha Malik, Duolao Wang, Samra Mazhar, Shahzad Alam Khan, Fareed Aslam Minhas, Atif Rahman

**Affiliations:** 1 Human Development Research Foundation (HDRF), Islamabad, Pakistan; 2 Department of Primary Care and Mental Health, University of Liverpool, Liverpool, UK; 3 Global Institute of Human Development, Shifa Tameer-e-Millat University, Islamabad, Pakistan; 4 Benazir Bhutto Hospital, Institute of Psychiatry, Rawalpindi Medical University, Rawalpindi, Pakistan; 5 London, UK; 6 Global Health Trials Unit, Liverpool School of Tropical Medicine, Liverpool, UK; 7 Ministry of National Health Services, Regulations and Coordination, Islamabad, Pakistan; 8 World Health Organization (WHO), Pakistan Office, Islamabad, Pakistan

**Keywords:** Adolescents, Psychosocial distress, Psychological interventions, Low resource school settings, School mental health, Feasibility, Cluster randomized control trial

## Abstract

**Background:**

Child and adolescent mental health problems are a global public mental health priority. However, there is a lack of evidence-based scalable psychological interventions for adolescents living in low resource settings. This trial was designed to evaluate the feasibility and acceptability of delivering the World Health Organization’s Early Adolescent Skills for Emotions (EASE) intervention at public schools in a rural sub-district in Rawalpindi, Pakistan.

**Methods:**

A two arm, single blinded, feasibility cluster randomized controlled trial with mixed-methods evaluation was conducted with 59 adolescents and their caregivers from 8 public schools. In the 4 intervention arm schools, 6 non-specialist facilitators delivered the culturally-adapted EASE group sessions to the adolescents (n = 29) and their caregivers with desired fidelity under the supervision of in-country supervisors.

**Results:**

The participation rate of adolescents in the intervention sessions was 83%. The intervention strategies were implemented by the adolescents. However, attending biweekly sessions at schools was challenging for caregivers with only 50% caregivers attending the sessions.

**Conclusions:**

The results of this study support the feasibility and acceptability of delivering this culturally adapted intervention through non-specialist facilitators in school settings in Pakistan and pave the way to conduct a fully powered cluster randomized controlled trial to test the effectiveness of intervention to improve psychological outcomes in adolescents.

*Trial registration* Trial registered with Clinicaltrials.gov prospectively; NCT04254393.

**Supplementary Information:**

The online version contains supplementary material available at 10.1186/s13034-022-00480-z.

## Background

Emotional conditions such as depression and anxiety are the leading contributors to health burden in adolescents worldwide [13, 50, 51]. Emotional difficulties in adolescents are predictive of mental health disorders in adulthood [37] and are associated with other adverse outcomes including academic failure, substance misuse and violence [14, 15]. Given that mental health problems affect one in five adolescents globally [16] and the rates of socio-emotional problems in adolescents are likely to exacerbate in the context of COVID-19 pandemic [27], there is an urgent need to address the ever growing mental health needs of adolescents.

The World Health Organization (WHO) has developed a brief, manualized trans-diagnostic, non-specialist delivered group psychological intervention, called Early Adolescent Skills for Emotions (EASE), a potentially scalable psychological intervention for adolescents with internalizing problems. The intervention consists of empirically supported strategies [10] and comprises of seven young adolescent group sessions and three-caregiver group sessions. Testing the feasibility and effectiveness of WHO EASE is taking place in many low resource and war affected settings including Jordan, Lebanon and Tanzania. Early findings suggest the intervention is acceptable in adolescents with psychological distress and their caregivers in refugee camps and schools of Tanzania [17] and for urban-residing Syrian refugees in Jordan [2].

Around 35% of Pakistan’s population is under the age of 14 years [47]. This population is particularly susceptible to mental health problems due to multiple co-occurring risk factors such as violence, political unrest, social and economic inequalities [22–24]. A recent epidemiological study, conducted with 5856 adolescents from 41 public schools in rural Pakistan reported 25% prevalence rate of psychosocial distress in school going adolescents [22–24]. Key barriers to the provision of evidence-based care for youth mental health in Pakistan include very limited budget allocation for mental health [22–24, 40, 52]; shortage of qualified and trained adolescent mental health experts; lack of evidence-based adolescent mental health programs and concentration of specialist adolescent mental health services to a few urban centres only.

Recognizing the lack of services and need for a holistic, public mental health approach to promote youth mental health in Pakistan, a Presidents’ Program to promote youth mental health through schools was launched in Oct, 2019. The program emphasises the role of school based, early life interventions to promote youth mental health in Pakistan [33]. We conducted this feasibility study as a part of the President’s Program. Before its implementation in public schools of Pakistan, we adapted the EASE intervention following the standard guidelines for translation and cultural adaptation of psychosocial interventions [3] consisting of the following steps; Step (1) literal translation of intervention content into Urdu. Since, the medium of instruction in public schools of Punjab province in Pakistan is ‘Urdu’—the national language of Pakistan, the EASE intervention materials including training manuals, storybooks and workbooks were translated in Urdu language and culturally adapted for implementation in public schools of Pakistan. During this step, cultural considerations were also taken into account in which expressions and content of intervention were also culturally adapted in consultation with local mental health experts. Step (2) back translation of Urdu version of the program into English in consultation with Master Trainer and mental health experts and Step (3) pre-piloting of the adapted program to finalize translation and adaptation: the draft version of the adapted intervention was pre-piloted with 15 Pakistani adolescents and their caregivers to ensure its cultural and contextual relevance. The findings of end-user testing informed the final version of the adapted EASE intervention for implementation in public schools of Pakistan. All cultural adaptation were recorded in the Bernal Framework and intervention specific develop adaptation log (publication forthcoming). The key research questions for the current feasibility study were: (a) is culturally adapted EASE acceptable to Pakistani young adolescents, their caregivers and education sector stakeholders and feasible for implementation in public schools of rural Pakistan? (b) Can non-specialist facilitators be trained and supervised using an apprenticeship model of training and supervision [34] and are they acceptable as delivery agents to young adolescents, their caregivers and education sector stakeholders for the delivery of EASE in school settings? (c) Assessment of the feasibility of trial procedures to inform the design of definitive cluster randomized controlled trial (cRCT), such as rates of recruitment, participation, retention at follow-up assessments and reliability of outcome measures. (d) What is the potential impact/safety of EASE intervention to improve psychological outcomes in adolescents with psychosocial distress?

## Methods

### Study design

Our research strategy was informed by the updated Medical Research Council (MRC) Framework for the development and evaluation of complex interventions which recognises the development of complex intervention in terms of non-sequential research phases including intervention development, its feasibility and piloting, effectiveness evaluation and implementation [43]. Having completed the translation and cultural adaptation of intervention for implementation in public schools of Pakistan, we conducted the feasibility evaluation of implementing the culturally adapted EASE intervention in public schools of Pakistan.

The present study was a two arm, single blinded, feasibility cluster randomized controlled trial with embedded mixed-methods process evaluation. Since, EASE is a group psychological intervention, we used a cluster randomized controlled trial design, where schools were randomized to intervention and control arms to avoid contamination between the two groups. The ethics approval for the study was obtained from the Ethics Review Committees of the University of Liverpool (UoL), UK and Human Development Research Foundation (HDRF), Pakistan. Only those participants (caregivers and adolescents) who provided written informed consent and met the trial eligibility criteria were included in the study. Prior to the commencement of program implementation, regulatory approvals were obtained from the School Education Department, Government of the Punjab and District Education Department, Rawalpindi.

### Settings and participants

The study was conducted in 8 public schools of the sub-district Gujar Khan, located in district Rawalpindi, Pakistan. The sub-district is representative of many other low- and middle-income sub-districts in Pakistan. Literacy rates in the study district are 80% [39]. The public-sector education system in Pakistan is organized in primary (up-to grade 5), middle (till grade 8) and high (from 9 to grade 10). There are 202 public schools (143-primary, 17-middle, and 42-high schools) in the study sub-district. For the purpose of the feasibility study, 8 middle and high public schools (4 boys’ and 4 girls’ schools), nominated for the feasibility study by District Education Department, were enrolled in the study and randomized following stratified randomization.

The study participants were recruited between December 2019 and January, 2020. Participants were male and female adolescents (aged 13–15) with psychosocial distress as assessed by Urdu version of self-reported Paediatric Symptoms Checklist (PSC) [22–24] and their caregivers.

Adolescents (a) with externalizing problems (i.e., screened positive, scored 7 and above on externalizing subscale of the PSC only); and/or (b) in need of acute protection (at high risk of abuse or harm to self or others) and/or (c) required immediate or ongoing medical or psychiatric care as reported by caregivers and participants themselves at the time of entry to the trial, were not included in the study. Safety measures were in place to ensure adolescents with imminent risks, identified at the time of enrolment, baseline and at follow-up assessments were referred to specialist mental health care facility.

### WHO Early Adolescent Skills for Emotions (EASE) intervention

The intervention comprises of seven group sessions for adolescents lasting 90 min and three group sessions for their caregivers, each lasting approximately 120-min. The intervention involves empirically supported components, see Table 1 for further detail on intervention strategies. The intervention material consists of (a) a facilitator’s manual to guide non-specialist facilitators through the implementation of each intervention session; (b) a story book to be used by the non-specialist facilitators to demonstrate how each intervention strategy can be implemented by an adolescent in his/her life; (c) posters to aid the intervention delivery by non-specialist facilitators and (d) a workbook that uses graphical illustrations to demonstrate intervention strategies to adolescents and helps them keep a record of intervention strategies practiced by them at home throughout the week. The progress of adolescents in implementing EASE intervention strategies is reviewed at the start of each subsequent group intervention session which includes the discussion on common problems faced by the adolescents in the implementation of intervention strategies and potential mitigation strategies to address these problems.

**Table 1:** Outline of WHO EASE adolescents and caregivers’ sessions, time-points and intervention strategies

Time-points	Sessions	Intervention strategies
Week 1	Youth session 1	Psychoeducation (understanding my feelings) Homework: participants try to identify as many feelings as they can each day and put them in the ‘Feelings Pots’ in their workbooks
Week 2	Youth session 2	Stress management (calming my body) Homework: the participants practice slow breathing once a day and shade or colour the balloon after they have practiced the slow breathing in their workbooks Participants keep identifying their feelings and fill the ‘feelings pot’ each day
Week 3	Caregiver session 1	Psychoeducation (understanding big and difficult feelings) Homework: active listening and quality time home practice
	Youth session 3	Behavioural activation (changing my actions part 1) Homework: participants complete their first planned step for ‘changing my actions’ using their staircase drawing Participants keep identifying their feelings and fill ‘feelings pot’ and colour ‘balloon’ after practicing slow breathing exercise each day
Week 4	Youth session 4	Behavioural activation (changing my actions part 2) Homework: participants complete their planned steps for ‘changing my actions’ using their staircase drawing Participants keep identifying their feelings and fill ‘feelings pot’ and colour ‘balloon’ after practicing slow breathing each day
Week 5	Youth session 5	Problem solving (managing my problems part 1) Homework: participants to complete their action plan for their best idea in Managing My Problems Participants keep identifying their feelings and fill ‘feeling pot’; colour ‘balloon’ after practicing slow breathing exercise each day and complete their plans for changing my actions in their workbooks
	Caregiver session 2	Praise (the power of praise) Homework: praise home practice
Week 6	Youth session 6	Problem solving (managing my problems part 2) Homework: participants complete their action plan for their new best idea in ‘Managing My Problems’ Participants keep identifying their feelings and fill ‘feeling pot’; colour ‘balloon’ after practicing slow breathing exercise each day and complete their complete plans for changing my actions
Week 7	Youth session 7	Relapse prevention (brighter futures)
	Caregiver session 3	Caregiver self-care and relapse prevention (brighter futures)

#### Training of non-specialist facilitators and in-country supervisors in WHO EASE intervention

In the present study, the delivery agents of the EASE intervention were non-specialist facilitators. They were male and females graduates with little or no prior experience of delivering psychological interventions. Non-specialist facilitators were supervised by in-country supervisors. In-country supervisors were mental health experts i.e., a psychiatrist and a psychologist, who were responsible for supervising the delivery of EASE intervention and ensured intervention fidelity. Eight facilitators and two in-country supervisors received 8 days’ classroom training (80–90 h) by the WHO master trainers. The training was conducted in-person in Pakistan. Intervention training included education on adversity and its impact upon mental health; training in basic counselling skills; managing distressed participants; delivering EASE intervention strategies and training in group facilitation; and facilitator self-care. The in-country supervisors received additional training in supervision skills by the master trainers. Before and after the training, the essential skills and competencies for delivering EASE were evaluated using an adapted Enhancing Assessment of Common Therapeutic factors (ENACT) rating scale [30] by the master trainers.

#### Field training of non-specialist facilitators

Following the classroom training, non-specialist facilitators delivered the intervention to a sample of adolescents and their caregivers selected from the local schools. A checklist-developed by intervention-developers was used to rate the fidelity and competency of non-specialist facilitators in delivering EASE. A random sample of 20% of sessions, delivered by each non-specialist facilitator, were directly observed by in-country supervisors. Non-specialist facilitators who performed satisfactorily on the ENACT scale (score 2 on each domain of the ENACT) during the classroom training and who passed the intervention specific competency and fidelity checks during the field training were selected for the role of EASE non-specialist facilitators for the feasibility trial.

#### Supervision of non-specialist facilitators during intervention delivery

Weekly supervision was provided to non-specialist facilitators by in-country supervisors (UH and ZeH); in turn, these supervisors were supported by a clinical supervisor who had been involved in the development of EASE (KD), on a fortnightly basis via video-conference call. Before each supervision meeting, non-specialist facilitators completed a supervision form that documented the challenges faced by the non-specialist facilitators during the intervention delivery. The supervision of non-specialist facilitators involved group discussion on (a) challenges faced by non-specialist facilitators while delivering intervention strategies; managing the group; managing individual participants or cases of risk and engaging with the school administration and (b) brainstorm potential mitigation strategies to address these challenges. A range of supervision techniques including group discussion; role plays; teaching and booster training sessions were used during supervision. Supervision of in-country supervisors by a master supervisor involved discussion of difficulties encountered in supervising the non-specialist facilitators, as well as self-care of supervisors and non-specialist facilitators.

#### Fidelity rating of non-specialist facilitators to deliver EASE

The intervention fidelity was assessed through rating a random sample of 15% of live observed sessions of each non-specialist facilitators by supervisors using an EASE intervention specific checklist that also included four additional items to assess group facilitation skills of non-specialist facilitators while delivering intervention sessions.

#### Delivery of EASE in school settings

An orientation session on EASE for the head teacher, teachers and school staff was organized at each school by the research team before EASE intervention delivery in schools. The objectives of orientation sessions were to share the interventional materials with school administration; mutually agree upon EASE intervention delivery schedule both for adolescents and caregivers’ sessions and to confirm the venues.

Six non-specialist facilitators (both male and female) delivered intervention in pairs in 4 intervention schools using the intervention manual. The group sessions were delivered to the adolescents on a weekly basis during the school hours in separate and quiet rooms in the schools to ensure privacy. The delivery of session involved introducing the session to participants; introducing an intervention strategy using the story book; and setting and reviewing home practice using the workbook. All sessions were delivered in Urdu.

### Wait-list control arm

Mental health services are not available in public schools of Pakistan to manage the mental health problems of children and adolescents; therefore, no structured programs were delivered to adolescents in the control arm schools. The results of the screening were shared with the participants of both study arms by the research team. The participants in both study arms were encouraged to seek specialist (psychologist/psychiatrist) support from the child and adolescents’ psychiatry department at the Institute of Psychiatry (IoP), the tertiary mental health care facility of the region. We maintained the record of health care services sought by study participants in both study arms using Client Service Receipt Inventory (CSRI) [7] at 3-months follow-up.

### Measures

#### Acceptability and feasibility of the EASE intervention

The acceptability and feasibility of EASE intervention was assessed through both quantitative and qualitative analysis. Feasibility was defined as the degree to which EASE has been successfully delivered within the school settings i.e., the extent to which it was feasible to train non-specialist facilitators, delivery of the intervention in school settings with fidelity; participation rates of adolescents and caregivers in intervention sessions and implementation of intervention strategies by the study participants. Acceptability is defined as participants and school education department’s experience with various aspects of the EASE intervention; their opinion about intervention structure and content and understanding of intervention strategies. It also included the level of ease with which the participants completed their homework practices and used the intervention workbook to implement intervention strategies in their daily routine.

The quantitative analysis involved assessments of non-specialist facilitators competency and fidelity of delivering the intervention; number of sessions delivered by non-specialist facilitators to study participants (to both adolescents and caregivers); duration of sessions delivered; and number of participants (both adolescents and primary caregivers) who received the intervention.

We conducted qualitative thematic analysis of the supervision records of non-specialist facilitators and participants’ intervention workbooks. The supervision record and participant’s workbooks contained the record of intervention strategies implemented by the study participants during the intervention sessions and home practice and challenges faced by the non-specialist facilitators during the intervention delivery.

#### Semi-structured interviews

The perceptions for the implementation in school settings was further assessed through targeted semi-structured interviews with study participants (n = 3) and non-specialist facilitators (n = 3). Interviews were audio recorded and transcribed for subsequent analysis.

#### Outcome measures

We evaluated the feasibility of using a range of psychological outcome measures with adolescents and their caregivers in the school settings including the suitability of outcome measures to evaluate potential impact of the adapted EASE intervention on mental health outcomes. The outcome measures were completed with the adolescent participants and their caregivers at two time points (at baseline and 3-months post-intervention) by trained research assessment team, blind to the allocation status of the schools. The baseline assessment was conducted in-person in school settings; whereas, 3-months endpoint assessment was conducted via telephone because of lockdown restrictions in the study sub-district due to COVID-19 pandemic.

As the primary aim of the EASE intervention is to reduce the symptoms of internalising disorders in adolescents (i.e., anxiety and depression), we proposed a reduction in psychosocial distress at 3-months post-intervention as our primary outcome of interest. We used the self-rated Paediatric Symptoms Checklist (PSC) [26] as a screening and outcome measure in our study. The youth version of Paediatric symptom checklist has 35-items and measures psychosocial distress including symptoms of externalizing, internalizing and attention problems with cut-offs of 7, 5, 7 respectively. Items are rated on a three-point Likert scale (0 = never, 1 = sometimes, 2 = often). Total score is calculated by summing the responses of all items. The tool has been translated into the Urdu language and validated in the same study district [22–24]. In the present study, previously validated cut-off score (PSC ≥ 28) of self-reported Urdu version of PSC was used [22–24]. We excluded the adolescents who screened positive on externalizing sub-scale only.

Secondary outcomes included: Patient Health Questionnaire (PHQ-9) [41] used for measuring depressive symptoms; somatic symptoms checklist to assess physiological symptoms of stress; the Social Problem-Solving Inventory—Revised Short Form [8] to assess functional and dysfunctional cognitive and emotional orientations towards solving problems; the Perceived Emotional/Personal Support Scale [44] for measuring perceived socio-emotional support received from family members, significant others and from friends; Short Warwick Edinburgh Mental Wellbeing Scale (SWEMWBS) [45] for measuring wellbeing; and Paediatric Quality of Life (PedsQL) for measuring adolescent quality of life [48].

The caregivers’ reported secondary outcomes included Paediatric Quality of Life (Peds-QL) Family impact module [48] to assesses caregivers’ health related quality and functioning and Alabama parenting scale [18] to measure five dimensions of parenting that are relevant to the aetiology and treatment of adolescents’ problems.

The cost of health services utilization at 3-months post-intervention was assessed with the adapted Client Services Receipt Inventory (CSRI) [7]. All instruments are validated and have been used previously in Pakistan or in similar settings [6, 21, 36].

### Randomization and blinding

An independent researcher, not involved in the study, randomized eight schools on 1:1 allocation ratio, stratified by gender, into intervention (n = 4) and control arm (n = 4) using computerized software. Allocation concealment was ensured by keeping the random assignments in sequentially numbered sealed envelopes. The Principal Investigator, assessment team and trial statistician were blind to the allocation status of trial participants. Blinding was ensured by instructing the participants to not disclose their allocation status during the assessments. Fidelity of blinding was assessed by having assessors guess the allocation status of participants at the end of assessments.

### Procedure

#### Stakeholder engagement

The ‘gatekeeper consenting bodies’ for the schools are the School Education Department, government of the Punjab and the District Education Department, Rawalpindi. Buy-in from these stakeholders is of paramount importance to implement any school-based interventions. As a part of our formative phase research, we did extensive needs assessment and stakeholder engagement using the Theory of Change (ToC) approach [19]. The ToC workshops were instrumental and served as a stakeholders’ engagement tool to develop collaborations most important to scaling-up school based mental health services in Pakistan [22–24].

#### Recruitment of trial participants

After obtaining written informed consent from the head teachers, parents and assent from adolescents of all participating schools, the paper based self-reported (Urdu version) Paediatric Symptoms Checklist (PSC) was administered to adolescents, aged 13–15 years in classroom settings. Adolescents, who screened positive for psychosocial distress on PSC (scored ≥ 28 on PSC total score) were evaluated against the eligibility criteria for participation in the trial. The baseline assessments with adolescents were conducted by trained assessment team members in the school settings, whereas assessments with the caregivers were completed at their home.

### Data analysis

The mixed-methods analysis consisted of qualitative data from intervention workbooks, supervision records and semi-structured interviews with adolescents and non-specialist facilitators. The qualitative data were analysed using thematic analysis approach. The thematic analysis moved through phases of familiarisation, generating codes, sub-themes and themes, reviewing themes, defining and naming themes and selecting illustrative quotes [4]. Data from non-specialist facilitators’ supervision record, participants’ intervention workbooks and semi-structured qualitative interview was triangulated to ensure a comprehensive understanding through convergence or divergence of findings relating to each category.

**Fig. 1:**
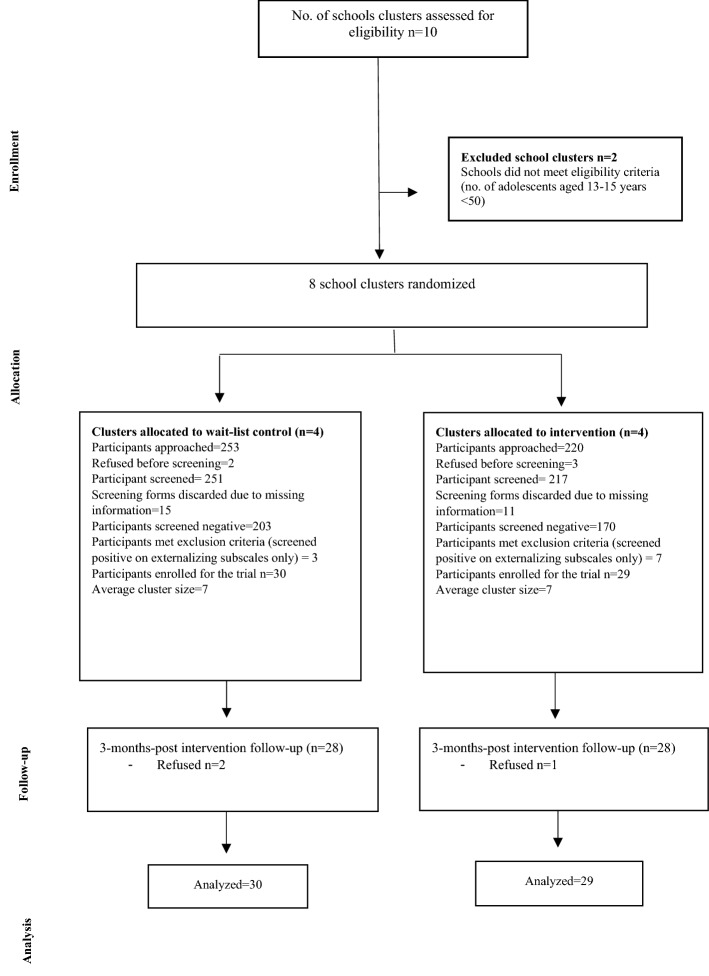
CONSORT flow diagram of the feasibility trial

Since this study was a feasibility cRCT, no power calculations were done; however, the sample was adequate to estimate the parameters for a planned definitive cRCT [5, 28]. With a sample size of 60 (30 per arm approximately), we aimed to generate some reliable estimate of potential treatment effect as well as recruitment and attrition rates (Fig. 1). The quantitative data was analysed on an intention-to-treat basis. Participants who dropped-out of the study were followed-up for endpoint assessment. A linear mixed model was employed for the primary and secondary outcome analyses, which had treatment as fixed effect, baseline measurement of the study outcome as covariate, and cluster as random effect. To control for potential confounding factors, a covariate adjusted linear mixed model analysis was performed on the following pre-specified covariates including age, gender and baseline severity of scores on PSC. The unadjusted and adjusted mean differences (AMD) between two treatment arms, together with its 95% confidence intervals (CIs), were derived from the mixed model. Missing data was treated as missing completely at random in the analyses. All analyses were performed on intention-to-treat basis and conducted using Statistical Analysis Software (SAS).

## Results

The findings of the study are reported following the updated recommendations of the CONSORT (CONsolidated Standards of Reporting Trials) 2010 statement: extension to feasibility randomized trials [12].

### Participant’s flow

Of the total of 473 potential adolescents, aged 13–15 years from 8 schools in the study sub-district, 69 participants screened positive for psychosocial distress. Ten participants met the exclusion criteria (screened positive on externalising subscales of PSC only). Fifty-nine participants (29 in intervention arm and 30 in control arm schools), meeting the eligibility criteria and their primary caregivers were included in the study. As soon as we completed the intervention delivery, the schools were closed due to COVID-19 pandemic. Therefore, we did not collect immediate-post intervention follow-up assessment data. To ensure the safety of participants and researchers amidst COVID-19, the primary-end-point data i.e., at 3-month post-intervention follow-up assessment, was collected via telephone. Three participants were lost to follow-up at the endpoint (see Fig. 1 for a detailed CONSORT flow diagram).

### Baseline characteristics of study participants

Table 2 shows the sample characteristics. The mean age of the adolescents was 13.59 (±0.77) years. 50% (30/59) of the adolescents were female. About 74% (44/59) of the adolescents were living in a nuclear family. All primary caregivers were women [96% (57/59) mothers]. The average monthly family income of adolescents was 12991.08 (± 15544.10) Pakistani rupees which is below the minimum monthly wage in Pakistan (i.e., 17000 PKR).

**Table 2:** Demographic characteristics (N = 59)

Characteristics	Total (N = 59)	Wait-list control (N = 30)	EASE intervention (N = 29)
Age (M, SD)	13.59 (0.77)	13.50 (0.68)	13.69 (0.85)
Gender			
Boys	29 (49.2%)	16 (53.3%)	13 (44.8%)
Girls	30 (50.8%)	14 (46.7%)	16 (55.2%)
Family structure (n, %)			
Nuclear	44 (74.6%)	20 (66.7%)	24 (82.8%)
Joint	15 (25.4%)	10 (33.3%)	5 (17.2%)
Mother’s education (n, %)			
None	5 (8.5%)	3 (10.0%)	2 (6.9%)
5 years of education	13 (22.0%)	5 (16.7%)	8 (27.6%)
6–10 years of education	33 (56%)	17 (56.6%)	16 (55%)
11 years of education and above	8 (13.5%)	5 (16.6%)	3 (10.3%)
Father’s education (n, %)			
None	11 (18.6%)	6 (20.0%)	5 (17.2%)
5 years of education	24 (40.7%)	15 (50.0%)	9 (31.0%)
6–10 years of education	18 (30.5%)	6 (20%)	12 (41%)
11 years of education and above	6 (10%)	3 (10%)	3 (10%)
Father’s employment			
Doctor	1 (1.7%)	0 (0%)	1 (3.4%)
Personal Business	19 (32.2%)	13 (43.3%)	6 (20.7%)
Manual Worker	14 (23.7%)	7 (23.3%)	7 (24.1%)
Does not work	5 (8.5%)	1 (3.3%)	4 (13.8%)
Other	20 (33.9%)	9 (30.0%)	11 (37.9%)
Monthly income in PKR (M, SD)	12,991.08 (15,544.10)	14,799.66 (14,087.31)	11,120.14 (16,965.46)

*M* mean, *SD* standard deviation

### Impact of adapted EASE intervention to reduce psychosocial distress in Pakistani adolescents

All outcome measures exhibited good psychometric properties as the alpha reliability of all tools ranged from 0.72 to 0.96. The average time to administer the complete set of outcome measures at baseline and follow-up assessment was 45 (± 23) minutes and 27(± 7) minutes with adolescents and caregivers, respectively. No change in the content or process of the administration of questionnaire was suggested by adolescents and their caregivers. Table 3 summarises results for the primary and secondary outcomes in the intervention and control arms on intention-to-treat analysis. At baseline, the intervention and control groups had similar scores on PSC total score [mean (SD) 34.17 (5.25) vs 34.52 (5.55)] as well as individual PSC subscales scores. At 3-months post-intervention, both intervention and control arms comparably reduced psychosocial distress on PSC total scores [mean (SD), 15.57 (10.28) vs 15 (10.42)], AMD, 0.42; 95% CI − 4.81 to 5.65) as well as on individual subscale scores and no difference in the score of PSC across two arms was observed. The covariate adjusted analysis shows difference between intervention and control groups in scores for the somatic symptoms, as measured by somatic symptoms checklist, AMD 1.61 (95% CI 0.26–2.96, *p* = 0.0207) and for depressive symptoms, as measured by Patient Health Questionnaire (PHQ-9) for adolescents, AMD 2.27 (95% CI 0.04–4.50, *p* = 0.0462) at 3-month post-intervention (Additional file 1: Table S1). There were no changes in either group for adolescents’ quality of life, emotional support, problem solving skills and caregivers’ quality of life (Additional file 1: Table S1). Because of lockdown imposed due to COVID-19 pandemic during the study period, the participants did not seek any health services during the study period.

**Table 3:** Summary of mixed model analysis of outcomes (N = 59)

Measurements	Visits	Descriptive statistics				Mixed model analysis	
		Wait list control		EASE Intervention		Difference in mean (95% CI)	p-value
		N	Mean (SD)	N	Mean (SD)		
PSC total score	Baseline	30	34.17 (5.25)	29	34.52 (5.55)		
	3-months post-intervention	28	15.57 (10.28)	28	15.00 (10.42)	0.42 (− 4.81 to 5.65)	0.8721
PSC internalizing score	Baseline	30	5.27 (1.80)	29	5.17 (1.69)		
	3-months post-intervention	28	1.57 (1.87)	28	1.79 (1.91)	− 0.33 (− 1.62 to 0.95)	0.6073
PSC externalizing score	Baseline	30	5.43 (2.50)	29	4.83 (1.97)		
	3-months post-intervention	28	2.64 (2.47)	28	2.11 (2.20)	0.33 (− 1.15 to 1.81)	0.6516
PSC attention score	Baseline	30	5.70 (1.86)	29	5.86 (2.12)		
	3-months post-intervention	28	2.75 (2.62)	28	2.43 (2.18)	0.28 (− 0.97 to 1.54)	0.6520
Somatic symptoms scores	Baseline	30	5.70 (3.55)	29	4.72 (2.74)		
	3-months post-intervention	30	3.18 (3.48)	28	1.89 (2.30)	1.10 (− 1.29 to 3.50)	0.3582
PHQ total score	Baseline	28	7.20 (5.06)	29	7.59 (3.91)		
	3-months post-intervention	30	5.64 (5.70)	28	3.71 (3.86)	2.11 (− 0.32 to 4.54)	0.0873
Perceived emotional personal support total score	Baseline	28	34.97 (17.76)	29	32.21 (14.23)		
	3-months post-intervention	30	25.43 (13.00)	28	29.56 (11.55)	− 4.12 (− 14.24 to 6.01)	0.4176
PEPSQ family subscale score	Baseline	28	16.43 (9.46)	29	18.03 (7.74)		
	3-months post-intervention	30	13.57 (5.46)	27	17.26 (7.36)	− 3.41 (− 7.86 to 1.05)	0.1307
PEPSQ others subscale score	Baseline	22	9.14 (5.48)	8	10.50 (1.69)		
	3-months post-intervention	9	9.22 (2.99)	7	9.14 (2.79)	− 1.05 (− 6.19 to 4.09)	0.6443
PEPSQ friends subscale score	Baseline	25	14.20 (7.11)	24	13.63 (5.17)		
	3-months post-intervention	16	15.56 (9.25)	20	13.40 (5.07)	0.73 (− 4.32 to 5.78)	0.7689
Edinburg Mental Wellbeing Scale total score	Baseline	30	18.67 (5.44)	29	20.52 (5.51)		
	3-months post-intervention	28	21.82 (5.50)	28	21.00 (5.22)	1.53 (− 1.25 to 4.30)	0.2744
Social problem solving inventory total score	Baseline	30	44.33 (13.31)	29	47.62 (9.93)		
	3-months post-intervention	28	37.46 (10.58)	28	35.75 (11.86)	2.54 (− 5.44 to 10.53)	0.5247
APQ total	Baseline	30	102.20 (19.51)	29	107.34 (17.53)		
	3-months post-intervention	28	107.50 (18.25)	28	106.07 (14.29)	3.64 (− 4.63 to 11.90)	0.3805
APQ positive parenting	Baseline	30	19.73 (4.32)	29	20.66 (4.72)		
	3-months post-intervention	28	21.32 (4.26)	28	22.04 (4.53)	− 0.28 (− 2.84 to 2.27)	0.8254
APQ involvement	Baseline	30	26.83 (6.93)	29	28.76 (6.99)		
	3-months post-intervention	28	1.68 (12.68)	28	51.79 (11.28)	0.85 (− 5.43 to 7.12)	0.7875
APQ poor monitoring	Baseline	30	19.87 (5.18)	29	20.66 (6.06)		
	3-months post-intervention	28	19.61 (6.27)	28	17.18 (4.92)	2.73 (− 0.10 to 5.55)	0.0582
APQ discipline	Baseline	30	12.80 (3.55)	29	13.17 (3.92)		
	3-months post-intervention	28	12.57 (3.95)	28	11.89 (3.10)	0.89 (− 1.32 to 3.11)	0.4214
APQ corporal punishment	Baseline	30	6.07 (1.72)	29	5.90 (2.13)		
	3-months post-intervention	28	5.86 (1.92)	28	5.07 (2.14)	0.75 (− 0.49 to 2.00)	0.2284
PPedsQL averaged out score	Baseline	30	79.65 (14.19)	29	81.54 (19.27)		
	3-months post-intervention	28	86.95 (14.71)	28	87.50 (13.45)	0.49 (− 9.79 to 10.77)	0.9240
PPedsQL physical functioning mean	Baseline	30	80.14 (17.97)	29	82.04 (20.02)		
	3-months post-intervention	30	88.36 (18.11)	28	91.52 (15.49)	− 2.26 (− 14.08 to 9.56)	0.7026
PPedsQL emotional functioning mean	Baseline	28	73.00 (17.65)	29	73.28 (24.97)		
	3-months post-intervention	30	80.86 (18.95)	28	84.31 (18.21)	− 3.15 (− 15.10 to 8.81)	0.5993
PPedsQL social functioning mean	Baseline	28	88.13 (14.71)	29	85.99 (21.24)		
	3-months post-intervention	30	87.72 (20.90)	28	89.66 (14.40)	− 2.78 (− 15.21 to 9.66)	0.6554
PPedsQL cognitive functioning mean	Baseline	28	82.00 (18.69)	29	80.34 (23.64)		
	3-months post-intervention	30	87.41 (18.45)	28	86.55 (18.13)	0.42 (− 12.64 to 13.47)	0.9488
PPedsQL communication mean	Baseline	28	83.06 (16.88)	29	85.63 (21.70)		
	3-months post-intervention	30	88.79 (15.63)	28	85.92 (20.06)	4.21 (− 8.06 to 16.47)	0.4937
PPedsQL worry mean	Baseline	28	67.17 (21.40)	29	78.62 (20.31)		
	3-months post-intervention	30	78.45 (21.01)	28	82.93 (15.21)	− 0.81 (− 18.78 to 17.15)	0.9280
PPedsQL daily activities mean	Baseline	28	78.33 (24.72)	29	83.62 (24.04)		
	3-months post-intervention	30	92.82 (13.68)	28	88.79 (18.67)	4.75 (− 5.50 to 15.00)	0.3565
PPedsQL family relations mean	Baseline	28	87.83 (13.43)	29	86.03 (22.93)		
	3-months post-intervention	30	94.14 (10.61)	28	89.83 (13.19)	3.73 (− 2.69 to 10.14)	0.2488
Ch PedsQL averaged out score	Baseline	28	60.98 (17.74)	29	65.78 (12.20)		
	3-months post-intervention	30	80.78 (15.92)	28	85.33 (13.79)	− 2.33 (− 10.44 to 5.79)	0.5666
Ch PedsQL physical funtioning mean	Baseline	28	65.63 (20.10)	29	67.35 (15.30)		
	3-months post-intervention		85.27 (16.79)	28	87.72 (14.28)	− 2.12 (− 11.15 to 6.92)	0.6398
Ch PedsQL averaged out score emotional functioning	Baseline	30	54.00 (22.26)	29	60.34 (17.32)		
	3-months post-intervention	28	75.71 (22.14)	28	81.79 (16.62)	− 3.65 (− 13.12 to 5.82)	0.4420
Ch PedsQL averaged out score social functioning	Baseline	30	63.50 (23.86)	29	68.62 (17.72)		
	3-months post-intervention	28	85.00 (15.99)	28	86.43 (19.04)	0.78 (− 11.47 to 13.03)	0.8987
Ch PedsQL averaged out score school functioning	Baseline	30	58.00 (20.20)	29	65.86 (13.76)		
	3-months post-intervention	28	74.46 (19.31)	28	83.93 (16.85)	− 4.85 (− 14.02 to 4.32)	0.2928

*PSC* Paediatric Symptoms Checklist; *PHQ* Patient Health Questionnaire; *PEPSQ* Perceived Emotional Personal Support; *APQ* Alabama Parenting Questionnaire; *PPedsQL* Paediatric Quality of Life Questionnaire-Family version; *Ch PedsQL* Paediatric Quality of Life Questionnaire-child reported

The allocation status of 12 (41%) of 29 participants in the intervention arm and 14 (46%) of 30 in wait-list control arm was correctly guessed by outcome assessors before the primary outcome assessment at 3-months post-intervention, indicating that blinding was successful.

### Competency and fidelity of non-specialist facilitators

We recruited 8 non-specialist facilitators for intervention delivery. Following the training and competency assessment, 6 non-specialist facilitators and 2 co-facilitators delivered training to trial participants. A total of 40 intervention sessions were delivered by 6 non-specialist facilitators in 4 intervention schools (28 youth and 12 caregivers’ sessions). The average group size was 7 and 4 for youth and caregivers’ sessions respectively. 25/29 (86%) of the trial participants attended all sessions. 16/29 (55%) caregivers attended all 3 sessions. Average duration of adolescents group session was 90 (± 10.5) minutes. Non-specialist facilitators and schools were gender matched to enhance acceptability. 20% (8/40) sessions were observed by in-country supervisors to assess the quality of intervention delivery. All non-specialist facilitators scored 95% and above on fidelity checklist. During the intervention delivery, seven weekly supervision meetings of non-specialist facilitators with in-country supervisors and three fortnightly supervision meetings of in-country supervisors (UH and ZeH) with master trainer (KD) were conducted.

### Feasibility and acceptability of delivering EASE intervention in schools

The adapted version of the intervention materials including facilitator manual, storybook and intervention workbook was formally reviewed and approved by the school education department, government of Punjab for implementation in public schools of Punjab province of Pakistan. The analysis of the intervention workbook data revealed that the major sources of stress for adolescents were academic pressure, family and peer problems. Majority of the adolescents reported using EASE intervention strategies to deal with their problems. The analysis of supervision data showed that most participants understood the intervention strategies and were able to use them appropriately. Most of the participants documented the use of the intervention strategies in their workbooks. The illustrative examples of intervention strategies implemented by study participants are given in Table 4.

**Table 4:** Thematic analysis of EASE workbook data and supervision notes of non-specialist facilitators on the use of EASE intervention strategies by adolescents

EASE Intervention strategies	Thematic analysis of EASE workbook on implementation of intervention strategies by adolescents	Thematic analysis of supervision notes of non-specialist facilitators
‘Understanding my feelings’ activity—identification of emotions	Identification of emotions 1. Felt anxious and worried because of academic problems (n = 15) 2. Sad, worried, angry and unhappy due to family problems/issues such as fights between parents, illness of caregiver and dispute with caregivers (n = 9) 3. Happy and angry while playing with friends (n = 4)	Level of understanding: most participants clearly understood the activity, identified multiple feelings and filled the feelings pot along with the key (n = 22) Difficulties: some participants faced difficulty in identifying their different feelings. Mostly they had only colored for sadness or happiness in their feeling pots (n = 2) Some participants had not drawn the key properly and sometimes it was missing and few participants lost interest in the strategy towards the end of the session (n = 4) A few participants completed home practice for the sake of completing it and could not relate to the activities (n = 3)
‘Calming my body’ activity**—**manage physical sensations while facing a difficult situation	Using managing physical sensations strategy 1. At night before falling asleep (n = 8) 2. While feeling angry, after having a fight with siblings and with friends (n = 3) 3. When felt physically hurt (n = 2) 4. When having physical sensations (increased heartbeat, palpitation, headache) while facing a difficult situation (n = 11) 5. When faced with bulling in the school (n = 4)	Level of understanding: most participant understood breathing exercise; practiced it while experiencing difficult emotions and understood the link between emotions and associated physical changes (n = 19) Difficulties: some participants faced difficulty in practicing slow breathing exercise initially (they reported dizziness and headache while practicing slow breathing exercise). (n = 4) A few participants had drawn similar drawing in all the body maps and could not link the emotions with physical sensations. (n = 3)
‘Changing my Actions’ activity	Use of changing my actions activity by boys: 1. Resuming playful activities (such as playing cricket, volley ball, football, marbles, badminton, carom board, kite flying) (n = 10) 2. Helping others (friends in studies, neighbours for carrying their groceries, parents in looking after domestic animals, siblings) (n = 4) 3. Hangouts with friends and cousins (n = 2) Use of changing my actions activity by girls: 1. Resuming playful activities (including hide and seek, reading storybooks, playing with sister, cousins and friends) (n = 4) 2. Household chores, cooking, and creative art activities (n = 8)	Level of understanding: most participants grasped the concept and appeared to understand the strategy well during the session and selected suitable activity for the strategy and, completed all steps. (n = 14) Difficulties: few participants were not able to think of any activities that they had stopped doing due to the big and difficult feelings. They only reported those activities that they had stopped doing due to lack of time and other external barriers (n = 9) A few participants struggled to understand the strategy however, with extra effort they were able to complete the steps of the activity (n = 2)
‘Managing my Problems’ activity	Use of managing my problems activity 1. To manage academic problems (getting late for school; procrastination, incomplete home work; poor hand writing; not preparing for test) (n = 9) 2. To manage problems with peers such as bullying; arguments and to stop fighting with friends, accusation of stealing from friends (n = 9) 3. To manage problems at home such as not doing home chores; conflict with siblings (n = 2) 4. To manage personal problems such as unable to sleep; finding time to play; forgetting things (n = 8)	Level of understanding: most participants understood the strategy well and were able to use the strategy to solve their problems (n = 18) Engagement: participants appeared interested and engaged particularly with this strategy. (n = 25) Difficulties: some participants had mentioned in their workbook that instead of trying one best solution, they had tried four solutions to solve their problem. (n = 3)

The analysis of the intervention workbook showed that both boys and girls opted for gender specific pleasurable and meaningful activities to overcome the feeling of distress (see Table 4).

Most adolescents used ‘problem solving’ activity to manage academic problems and peer problems.

“*Due to (my habit of) procrastination, I always find it difficult to prepare for tests, but managing my problems activity helped me to make a time table which helped me in managing my school work.”* (P8)

All study participants were able to relate with the content of the storybook and enjoyed practicing intervention strategies using the workbook. The details on implementation of each intervention strategies by adolescents including reflections of non-specialist facilitators are mentioned in Table 4.

The analysis of supervision record showed that caregivers appreciated the concept of spending quality time with their children, however, ‘praising their children’ was relatively a new concept for them.

*“Previously I used to think that I should not praise my child as he might get spoiled and not obey me. But now I learned how important it (praise) is to build his self-confidence.” (C-1, quote from EASE caregivers’ session)*

The EASE intervention sessions increased caregivers’ knowledge about adolescent’s psychosocial distress and they found intervention strategies to be useful in helping their children. Moreover, welcoming environment of the schools and group learning motivated them to participate in the intervention sessions. During the sessions, some caregivers felt uncomfortable due to the language barrier (as some of the caregivers were not well familiar with the national language, Urdu and were comfortable in speaking in their local language i.e., Pothwari) and in performing some group activities such as role plays. Consequently, some of the caregivers did not open-up and share their experiences with the group. Other barrier for caregivers to attend the intervention sessions were challenges in traveling to the schools; competing demands on their time and health problems.

Delivering the culturally adapted, manual based intervention was feasible for the non-specialist facilitators. Adequate support from in-country supervisors, written description of the intervention content and layout of the sessions and presence of a co-facilitator were key factors reported by the non-specialist facilitators that contributed to the quality of intervention delivery.

According to the non-specialist facilitators, buy-in from school administration enhanced the feasibility and acceptability of implementing EASE intervention in the schools. The school administration expressed their trust on the content of the intervention, provided adequate time and venue to deliver both adolescents and caregivers sessions in the school settings (see Table 5).

**Table 5:** Thematic analysis of semi-structured interviews (N = 6) to evaluate implementation EASE intervention in public schools of Pakistan

Objectives	Themes	Illustrative quotes
Acceptability to adolescents	Better able to cope with distress	*“Previously, I used to get angry at my sister but yesterday I practiced slow breathing exercise when I felt anger at my sister and it helped me to control my anger.” *(Youth) *“Whenever my heart beat goes up, I practice breathing exercise and feel relaxed.” *(Youth) *“I fought with my friend, and felt sad after some time. Then I used the ‘tree exercise’ to resolve the problem.” *(Youth)
	Helpful strategies to resolve daily life problems	*“I used to get late for school and I was having difficulty in managing time but after learning problem solving strategy in session and applying it, my problems has been resolved.” *(Youth) *“These sessions/strategies helped me in my school work.” *(Youth) *“If I ever receive a chance to be a part of these sessions again, I would be willing to attend this program.” *(Youth)
	Ensuring confidentiality	*“As you (non-specialist facilitators) keep my confidentiality intact, so I feel comfortable in sharing my personal problems with you.”* (Youth) *“It is good that our conversations will stay within this group.”* (Youth)
	Relatable story	*“I really like to hear Noor’s Story from you as I find it quite relatable to my daily life problems and it gives me confidence that I can solve my problems too.”* (Youth) *“Noor’s story is really interesting.”* (Youth) *“I like the example in which Noor spots birds.”* (Youth)
	Peer pressure	*“Other students say to me that you are missing out your studies because of these sessions and these sessions are useless.” *(Youth) *“Some students in the van pressurize me into sharing all about the sessions, and I was not comfortable with it.” *(Youth) *“The other day one of my class fellows wanted to see my workbook, but I did not want to show it to him.” *(Youth)
Acceptability to caregivers	Increase in knowledge about parenting	*“It is very helpful to learn useful strategies from them (non-specialist facilitators) and I really want to thank them for coming here for us and our children. I would like to attend such sessions in future as well.” *(Caregiver) “*Previously I didn’t praise my child as it might spoil him but after attending this session I realized the importance of praise and I even praise him for his small efforts.” *(Caregivers) *“My child felt very happy when I actively listened to him.” *(Caregiver)
	Welcoming session environment	*“We really like to come here for our children and you welcome us so nicely.” *(Caregiver) *“It is great to see that you care for our children, and engage us too.” *(Caregiver) *“Your positive behavior motivates me to attend the session.” *(Caregiver)
	Group learning	*“I have learnt a lot from the experiences of others (parents) in the group.” *(Caregiver) *“The group sessions have helped me better understand my child’s problems.” *(Caregiver) *“It was difficult for me to understand the components of praising the child, but with the support of this I was able to learn this.” *(Caregiver)
	Traveling issue	*“We get late in the sessions because it is challenging to find a transport at this time and we have to travel a long distance.” *(Caregiver) *“I cannot travel alone and need someone to accompany me for the session. I came with my daughter today therefore I couldn’t send her to school.” *(Caregiver) *“Since I have a health problem, it is not easy for me to cover long distance on foot.” *(Caregiver)
	Personal commitment	*“It is hard to attend session along with household activities.” *(Caregiver) *“My working hours are being compromised because of attending the session.” *(Caregiver) *“Normally I am supposed to complete my household chores around this time.” *(Caregiver)
	Language Barrier	*“We were unable to understand the intervention strategies completely, would it be possible for you to deliver the key concepts in our local language?”* (Caregiver) “*It will be easy for us to participate in discussion in our local language.”* (Caregiver)
Acceptability to non-specialist facilitators	Helpful strategies for adolescents	*“At the beginning, it was challenging for the children to open-up however, with the passage of time we developed a trustful relationship and children were more comfortable in sharing their stressful experiences with us.” *(non-specialist facilitators) *“Children were able to practice slow breathing exercise to manage their difficult feelings due to repeated demonstration of this strategy during the session.” *(non- specialist facilitators) *“It was satisfying to see the children feel comfortable sharing their thoughts and problems in group settings.” *(non-specialist facilitators)
	Cooperation from schools	*“School staff was very cooperative in terms of providing proper venue and ensuring the availability of children for the sessions and they coordinated with the parents as well.” *(non-specialist facilitator) *“Well, the school administration was quite aware of the President’s Mental Health Program, and they welcomed us wholeheartedly.” *(non-specialist facilitators) *“Some of the school principals showed great interest in our work with children and had post-session discussions with non- specialist facilitators to discuss how best children can be supported in their development and academics.” *(non-specialist facilitators)

The non-specialist facilitators reported that the implementation of EASE intervention in the school settings resulted in increased awareness of school staff in the importance of adolescents recognizing and managing their emotions. The school staff held debrief discussions with the non-specialist facilitators to explore potential barriers in the delivery of the sessions such as low attendance of parents in the caregiver sessions and encouraged caregivers to attend the sessions. Moreover, the schools also compensated the academic loss of adolescents who attended the EASE intervention sessions by pairing them with their peers.

*“Some of the school principals showed great interest in our work with children and had post-session discussions with non- specialist facilitators to discuss how best children can be supported in their development and academics.” *(non-specialist facilitator)

Apart from the supervision notes and workbook data, we conducted 3 in-depth interviews with adolescents and 3-in-depth interviews with non-specialist facilitators to explore the facilitators and barriers of implementing the EASE intervention in school settings. As the post-program qualitative interviews were conducted amidst COVID-19 pandemic, these interviews were conducted telephonically. The findings of in-depth interviews showed that the EASE intervention was acceptable to all adolescents as it helped them to cope with feelings of distress and resolve daily problems including academic problems as well as relationship problems. Also, they were able to relate with the content of the intervention and developed a good rapport with the non-specialist facilitators. Ensuring confidentiality helped them to open-up and share their personal experiences without the fear of being judged during the sessions. For adolescents, the most commonly reported barrier was the fear of stigmatization from other students because of attending the EASE intervention sessions in school settings (Table 5). No adverse life event was reported during intervention delivery or at post-intervention follow-up.

## Discussion

The present study evaluated the acceptability and feasibility of delivering adapted EASE intervention in public schools of rural Rawalpindi in Pakistan. The results of the feasibility trial showed that non-specialist facilitators can be trained to deliver the EASE intervention with desired competency and fidelity in school settings under the supervision of in-country supervisors. The non-specialist facilitators were acceptable to adolescents, caregivers and school administration as delivery agents of group psychological intervention. The adolescents found the intervention strategies to be helpful and used these strategies to deal with their academic, family and peer problems. The feasibility testing of research procedures indicated successful recruitment of eligible adolescents; implementation of intervention activities in public school settings; and feasibility and acceptability of conducting trial assessments in class rooms by trained assessment team and through telephone in the post-COVID-19 context. All outcome measures exhibited good psychometric properties. At 3-months post-intervention, reduction in the PSC scores was observed in both study arms. The possible reason could be due to the regression toward the mean phenomena that might have contributed in equally huge reduction in the score of control arm similar to the intervention arm. Studies show that natural remission seems to be more frequent in mild to moderate cases of emotional problems, especially in the sample of adolescents [50, 51]. These findings contribute to the existing knowledge base on the feasibility of implementing trans-diagnostic, task-shifting, psychological interventions to address emotional problems in adolescents in low resource school settings [14, 15].

The qualitative analysis of the data demonstrated that the adapted EASE intervention resulted in an increased emotional awareness among adolescents and they were able to identify feelings of worry, sadness, anger and happiness as a result of everyday life events. Academic pressures, family and peer problems were the main sources of distress for them. To deal with the physical symptoms of distress, they used breathing exercise and resumed culturally-gender specific pleasurable and meaningful activities to overcome their feelings of distress. They effectively learned how to implement the 5 steps of problem-solving intervention strategy to manage their problems. The strategies included in EASE are empirically supported for emotional problems in school going adolescents, especially living in low resource settings. In a study of 248 Indian adolescents in need of psychological support, the problem solving intervention strategy was found to be helpful to not only reduce the emotional problems; but, also to manage the practical problems of adolescents [20]. Similarly, there is emerging evidence that trans-diagnostic behavioural activation (which targets the avoidance behaviour of adolescents to participate in pleasurable and meaningful activities because of their feeling of distress) and stress management strategies (mainly breathing exercise) are effective to improve both anxiety and depressive symptoms in adolescents [31]. Although our study was not powered to detect the statistically significant difference between two groups on psychological outcomes, the covariate adjusted analysis showed statistically significant reduction in depressive and somatic distress symptoms in intervention arm, compared to control arm at 3-months post-intervention. Studies showed that targeted, cognitive behavioural therapy based group interventions, compared to community based programs, are more effective in reducing depressive symptoms and anxiety in adolescents [9]. Group psychological interventions can be an efficient way to increase the access to psychological care for adolescents with emotional problems, especially in low resource settings [42].

Involvement of parents in the management of emotional problems of adolescents is one of the most empirically supported intervention strategies [56]. A key component of EASE intervention was to help parents/caregivers improve active listening skills and emotion identification in themselves and in their children; increasing the quality time spent with their children, an increase in praise given to their children and increased implementation of self-care strategies by parents/caregivers. In the present study, only 50% of the sample of caregivers received the full dose of intervention. Thematic analysis of supervision record revealed that it was challenging for caregivers to attend biweekly intervention sessions at schools. Poor attendance of caregivers in mental health programs has been identified as one of the most significant barriers to deliver the mental health programs in LMICs [32, 35]. Studies have shown that in the community based treatment programs, with an average duration of three to five sessions, maintaining the adequate attendance of the caregivers becomes challenging [25]. A number of barriers have been reported that could affect the participation of caregivers into the program, including the cost, transportation challenges, competing demands on the time of caregivers, low acceptability of the intervention and mental health stigma [38]. Similarly, in our study, transportation challenges and competing demands on caregivers’ time and health problems created hindrance for them to participate in the intervention sessions. Addressing these challenges becomes imperative to ensure effective involvement of caregivers in adolescent mental health program. These challenges could be addressed by organizing the caregivers’ intervention sessions at convenient place, i.e., near to the caregivers’ work or home or by providing monetary incentives for transportation and compensation of their time in attending the sessions. Despite the fact that these strategies could help to improve caregivers’ participation into the sessions, these might have budget implications and pose challenges to the sustainability of such programs. There is evidence that the partnership between health and academic institutes can improve the access of youth mental health services in low resource settings, globally [1]. Supportive role of school teachers could also help to improve the attendance of caregivers in the treatment sessions [32], provided there is buy-in from the school staff including school leadership, administration and teachers to implement the school based mental health programs.

Our study demonstrates the feasibility of delivering intervention by non-specialist facilitators in school settings. In our study, we used a cascade model of training and supervision [34] to train university graduate students with no prior experience in delivering mental health services. In Low- and Middle-Income Countries (LMICs) shortage of trained human resources in the field of child and adolescent mental health is well established. Community based programs that use task shifting approaches by engaging non-specialists including community health workers, nurses, teachers and graduate students can be effectively used to deliver quality mental health services at a lower cost, through the existing platforms under the supervision of mental health specialists [29, 53]. It is one of the most promising strategies to mitigate the scarcity of human resource in the field of mental health and has been proven to be effective in providing mental health services to the people in resource poor areas, globally [11].

One of the key strengths of our study was high participation rate in youth sessions which can be attributed to delivering the intervention in a more structured environment like schools. Schools have been identified as a potential platform to reach unprecedented number of children and adolescents and to deliver promotive, preventive and indicated mental health intervention and wellbeing programs to promote youth mental health [49, 55], especially in low resource settings. In Pakistan, reducing the treatment gap for child and adolescent mental health in vulnerable populations including the adolescents in low resource public schools who are exposed to a multitude of chronic adversities such as poverty, illiteracy and unemployment, is a national priority [33, 54]. The President’s program to promote youth mental health through schools provided an opportunity to feasibility test the non-specialist delivered WHO Early Adolescent Skills for Emotions (EASE) intervention in public schools of Pakistan. Leadership from the highest office of the President of Pakistan to promote youth mental health, developing formal collaborations between the Ministry of Health, School Education Department, national and international academic and implementation partners and the use of a World Health Organization endorsed, empirically supported intervention were instrumental to the successful implement the program in the public schools [22–24].

The findings of the current feasibility study have important implications to guide the research procedures for the planned definitive cluster randomized controlled trial (cRCT) in a suitable direction. In the feasibility study, the EASE intervention was branded as an emotional skills training workshop for adolescents to avoid the stigma of implementing mental health intervention to a specific group of adolescents with psychosocial distress in school settings. Using the trans-diagnostic EASE intervention, which avoided the need for a specific diagnosis and risk of labelling and stigmatization, further helped to improve the intervention acceptability among the relevant stakeholders. The trial procedures including recruitment, intervention implementation and follow-up assessment (especially conducting telephonic assessments to minimize the risk of harm to participants and researchers in the on-going COVID-19 pandemic) have been feasibility tested prior to conducting the fully powered cRCT in school settings and these procedures are likely to be replicated without any rigorous methodological changes. Moreover, the feasibility trial helped further strengthen the partnerships and collaborations between health and education department that are important for implementing any school-based interventions. An important implication that warrants attention is the selection of psychological outcome measures for impact evaluation of the EASE intervention in the definitive cRCT. While the Paediatric Symptoms Checklist demonstrated good psychometric properties as a screening instrument to identify adolescents with psychosocial distress [22–24], the reduction in the scores of somatic and depressive symptoms at 3-months post-intervention encourage a careful consideration of using symptoms specific clinical outcome measures such as Revised Children Anxiety and Depression scale as the primary outcome of interest for the planned definitive cRCT [46].

### Limitation

While we were successful in training non-specialist facilitators to deliver WHO EASE intervention in public schools of rural Rawalpindi in Pakistan, a key limitation in our study is that we could not evaluate the immediate impact of the intervention due to COVID-19 pandemic and closure of schools. This can be mitigated in the planned definitive cRCT by moving from face-to-face to telephonic assessments to ensure safe data collection during on-going COVID-19 pandemic. Another limitation of our study was that we did not conduct qualitative in-depth interviews with caregivers rather analysed the supervision records to explore caregivers’ experience of receiving EASE. Although actual verbatims of the caregivers that they shared during the EASE sessions were thematically analysed, there might be a bias of non-specialists to only record the positive experience of caregivers that were in the favour of intervention and share it during supervision meetings. In this context, in-depth exploration of caregivers’ experience of receiving intervention in definitive cRCT using mixed methods design become important. Lastly, there was 50% attendance of caregivers in intervention sessions due to a number of challenges including traveling and personal commitments which created hindrance for them to participate in the intervention sessions. In future these challenges could be addressed by moving the location of caregivers’ sessions to be more convenient (e.g., near the caregivers' work or home) or by providing monetary incentives for travel.

### Conclusions

The findings of our study support the feasibility and acceptability of delivering culturally adapted intervention by non-specialist facilitators in school settings of Pakistan and lead to a fully powered cluster randomized controlled trial to test the effectiveness of intervention to improve psychological outcomes in adolescents in low resource school settings.

## Supplementary Information

**Additional file 1: Table S1.** Summary statistics and results from mixed model analysis of outcomes: covariate adjusted analysis (n = 59).

## Data Availability

The dataset generated and analysed during the current study are available from the corresponding author on reasonable request.

## Abbreviations

EASE: Early Adolescent Skills for Emotions
cRCT: Cluster randomized controlled trial
WHO: World Health Organization
MRC: Medical Research Council
UoL: University of Liverpool
HDRF: Human Development Research Foundation
PSC: Paediatric Symptoms Checklist
ENACT: Enhancing assessment of common therapeutic factors
IoP: Institute of Psychiatry
CSRI: Client Service Receipt Inventory
PHQ-9: Patient Health Questionnaire-9
SWEMWBS: Short Warwick Edinburgh Mental Wellbeing Scale
PedsQL: Paediatric Quality of Life
APQ: Alabama Parenting Questionnaire
ToC: Theory of change
CI: Confidence interval
SAS: Statistical Analysis Software
CONSORT: CONsolidated Standards of Reporting Trials
PKR: Pakistani rupees
AMD: Adjusted mean differences
LMICs: Low- and middle-income countries
