# Supplementary material for: Feasibility and acceptability of a multicomponent, group psychological intervention for adolescents with psychosocial distress in public schools of Pakistan: a feasibility cluster randomized controlled trial (cRCT)
Source: Child Adolesc Psychiatry Ment Health. 2022 Jun 21;16:47. doi: 10.1186/s13034-022-00480-z (PMC9210054; doi:10.1186/s13034-022-00480-z)
Supplement: Supplementary file 1 — Additional file 1: Table S1. Summary statistics and results from mixed model analysis of outcomes: covariate adjusted analysis (n = 59). [file 13034_2022_480_MOESM1_ESM.docx]

**Additional file**

**Table S1. Summary statistics and results from mixed model analysis of outcomes: covariate adjusted analysis (n=59)**

| **Primary and secondary outcomes** | **Measurements** | **Visit** | **Descriptive statistics** | | | | **Mixed model analysis** | |
| --- | --- | --- | --- | --- | --- | --- | --- | --- |
|  |  |  | **N** | **Wait list control** | **N** | **Intervention** | **Difference (95%CI)** | **p-value** |
|  |  |  |  | Mean (SD) |  | Mean (SD) |  |  |
| Paediatrics Symptoms Checklist | PSC Total Score | Baseline | 30 | 34.17(5.25) | 29 | 34.52(5.55) |  |  |
|  |  | 3 Months | 28 | 15.57(10.28) | 28 | 15.00(10.42) | 0.64(-4.42,5.70) | 0.8003 |
|  | PSC Internalizing Score | Baseline | 30 | 5.27(1.80) | 29 | 5.17(1.69) |  |  |
|  |  | 3 Months | 28 | 1.57(1.87) | 28 | 1.79(1.91) | -0.18(-1.15,0.78) | 0.7011 |
|  | PSC Externalizing Score | Baseline | 30 | 5.43(2.50) | 29 | 4.83(1.97) |  |  |
|  |  | 3 Months | 28 | 2.64(2.47) | 28 | 2.11(2.20) | 0.35(-0.85,1.55) | 0.5569 |
|  | PSC Attention Score | Baseline | 30 | 5.70(1.86) | 29 | 5.86(2.12) |  |  |
|  |  | 3 Months | 28 | 2.75(2.62) | 28 | 2.43(2.18) | 0.45(-0.73,1.62) | 0.4500 |
| Physiological symptoms scores | Physiological symptoms scores | Baseline | 30 | 5.70(3.55) | 29 | 4.72(2.74) |  |  |
|  |  | 3 Months | 28 | 3.18(3.48) | 28 | 1.89(2.30) | 1.61(0.26,2.96) | 0.0207 |
| Patient Health Questionnaire 9 | PHQ Total Score | Baseline | 30 | 7.20(5.06) | 29 | 7.59(3.91) |  |  |
|  |  | 3 Months | 28 | 5.64(5.70) | 28 | 3.71(3.86) | 2.27(0.04,4.50) | 0.0462 |
| Perceived Emotional Personal Support Questionnaire | Perceived Emotional Personal Support Total Score | Baseline | 30 | 34.97(17.76) | 29 | 32.21(14.23) |  |  |
|  |  | 3 Months | 28 | 25.43(13.00) | 28 | 29.56(11.55) | -3.85(-15.17,7.48) | 0.4972 |
|  | PEPSQ Family subscale score | Baseline | 30 | 16.43(9.46) | 29 | 18.03(7.74) |  |  |
|  |  | 3 Months | 28 | 13.57(5.46) | 27 | 17.26(7.36) | -3.57(-8.16,1.02) | 0.1241 |
|  | PEPSQ Others subscale score | Baseline | 22 | 9.14(5.48) | 8 | 10.50(1.69) |  |  |
|  |  | 3 Months | 9 | 9.22(2.99) | 7 | 9.14(2.79) | 0.16(-6.63,6.95) | 0.9522 |
|  | PEPSQ Friends subscale score | Baseline | 25 | 14.20(7.11) | 24 | 13.63(5.17) |  |  |
|  |  | 3 Months | 16 | 15.56(9.25) | 20 | 13.40(5.07) | -0.16(-4.34,4.02) | 0.9367 |
| Edinburgh Mental Wellbeing Scale | Edinburg Mental Wellbeing Scale Total Score | Baseline | 30 | 18.67(5.44) | 29 | 20.52(5.51) |  |  |
|  |  | 3 Months | 28 | 21.82(5.50) | 28 | 21.00(5.22) | 1.29(-1.57,4.16) | 0.3687 |
| Social Problem Solving Inventory | Social Problem Solving Inventory Total Score | Baseline | 30 | 44.33(13.31) | 29 | 47.62(9.93) |  |  |
|  |  | 3 Months | 28 | 37.46(10.58) | 28 | 35.75(11.86) | 3.03(-4.21,10.27) | 0.4037 |
| Alabama Parenting Questionnaire | APQ Total | Baseline | 30 | 102.20(19.51) | 29 | 107.34(17.53) |  |  |
|  |  | 3 Months | 28 | 107.50(18.25) | 28 | 106.07(14.29) | 2.35(-6.84,11.54) | 0.6090 |
|  | APQ positive parenting | Baseline | 30 | 19.73(4.32) | 29 | 20.66(4.72) |  |  |
|  |  | 3 Months | 28 | 21.32(4.26) | 29 | 22.04(4.53) | -0.53(-3.25,2.19) | 0.6968 |
|  | APQ involvement | Baseline | 30 | 26.83(6.93) | 28 | 28.76(6.99) |  |  |
|  |  | 3 Months | 28 | 51.68(12.68) | 29 | 51.79(11.28) | 0.25(-6.79,7.28) | 0.9438 |
|  | APQ poor monitoring | Baseline | 30 | 19.87(5.18) | 28 | 20.66(6.06) |  |  |
|  |  | 3 Months | 28 | 19.61(6.27) | 29 | 17.18(4.92) | 2.47(-0.43,5.38) | 0.0926 |
|  | APQ discipline | Baseline | 30 | 12.80(3.55) | 28 | 13.17(3.92) |  |  |
|  |  | 3 Months | 28 | 12.57(3.95) | 29 | 11.89(3.10) | 0.74(-1.45,2.93) | 0.4994 |
|  | APQ Corporal Punishment | Baseline | 30 | 6.07(1.72) | 28 | 5.90(2.13) |  |  |
|  |  | 3 Months | 28 | 5.86(1.92) | 29 | 5.07(2.14) | 0.76(-0.42,1.94) | 0.2004 |
| Paediatric Quality of Life (Family Impact Module) | PPedsQL averaged out score | Baseline | 30 | 79.65(14.19) | 28 | 81.54(19.27) |  |  |
|  |  | 3 Months | 28 | 86.95(14.71) | 29 | 87.50(13.45) | 0.21(-11.15,11.57) | 0.9703 |
|  | PPedsQL physical functioning Mean | Baseline | 30 | 80.14(17.97) | 28 | 82.04(20.02) |  |  |
|  |  | 3 Months | 28 | 88.36(18.11) | 29 | 91.52(15.49) | -2.46(-15.10,10.18) | 0.6969 |
|  | PPedsQL emotional functioning Mean | Baseline | 30 | 73.00(17.65) | 28 | 73.28(24.97) |  |  |
|  |  | 3 Months | 28 | 80.86(18.95) | 29 | 84.31(18.21) | -3.00(-16.95,10.95) | 0.6671 |
|  | PPedsQL social functioning Mean | Baseline | 30 | 88.13(14.71) | 28 | 85.99(21.24) |  |  |
|  |  | 3 Months | 28 | 87.72(20.90) | 29 | 89.66(14.40) | -3.31(-17.10,10.49) | 0.6316 |
|  | PPedsQL cognitive functioning Mean | Baseline | 30 | 82.00(18.69) | 28 | 80.34(23.64) |  |  |
|  |  | 3 Months | 28 | 87.41(18.45) | 29 | 86.55(18.13) | -0.14(-14.26,13.99) | 0.9846 |
|  | PPedsQL communication Mean | Baseline | 30 | 83.06(16.88) | 28 | 85.63(21.70) |  |  |
|  |  | 3 Months | 28 | 88.79(15.63) | 29 | 85.92(20.06) | 3.64(-8.58,15.85) | 0.5520 |
|  | PPedsQL worry Mean | Baseline | 30 | 67.17(21.40) | 28 | 78.62(20.31) |  |  |
|  |  | 3 Months | 28 | 78.45(21.01) | 29 | 82.93(15.21) | -1.85(-20.10,16.40) | 0.8389 |
|  | PPedsQL daily activities Mean | Baseline | 30 | 78.33(24.72) | 28 | 83.62(24.04) |  |  |
|  |  | 3 Months | 28 | 92.82(13.68) | 29 | 88.79(18.67) | 4.16(-6.42,14.73) | 0.4328 |
|  | PPedsQL family relations Mean | Baseline | 30 | 87.83(13.43) | 28 | 86.03(22.93) |  |  |
|  |  | 3 Months | 28 | 94.14(10.61) | 29 | 89.83(13.19) | 3.91(-2.02,9.84) | 0.1913 |
| Pediatric Quality of Life (Child Version) | Ch PedsQL averaged out score | Baseline | 30 | 60.98(17.74) | 28 | 65.78(12.20) |  |  |
|  |  | 3 Months | 28 | 80.78(15.92) | 29 | 85.33(13.79) | -4.26(-11.54,3.02) | 0.2443 |
|  | Ch PedsQL Physical Funtioning Mean | Baseline | 30 | 65.63(20.10) | 28 | 67.35(15.30) |  |  |
|  |  | 3 Months | 28 | 85.27(16.79) | 29 | 87.72(14.28) | -2.95(-10.95,5.05) | 0.4616 |
|  | Ch PedsQL averaged out score Emotional Functioning | Baseline | 30 | 54.00(22.26) | 28 | 60.34(17.32) |  |  |
|  |  | 3 Months | 28 | 75.71(22.14) | 29 | 81.79(16.62) | -5.28(-15.00,4.45) | 0.2800 |
|  | Ch PedsQL averaged out score Social Functioning | Baseline | 30 | 63.50(23.86) | 28 | 68.62(17.72) |  |  |
|  |  | 3 Months | 28 | 85.00(15.99) | 29 | 86.43(19.04) | -1.63(-9.50,6.25) | 0.6794 |
|  | Ch PedsQL averaged out score School Functioning | Baseline | 30 | 58.00(20.20) | 28 | 65.86(13.76) |  |  |
|  |  | 3 Months | 28 | 74.46(19.31) | 29 | 83.93(16.85) | -6.97(-16.60,2.65) | 0.1514 |

**Abbreviation:** PSC, Paediatric Symptoms Checklist; PHQ, Patient Health Questionnaire; PEPSQ, Perceived Emotional Personal Support; APQ, Alabama Parenting Questionnaire; PPedsQL, Paediatric Quality of Life Questionnaire-Family version; Ch PedsQL, Paediatric Quality of Life Questionnaire-child reported
